# Exploring the feasibility of an exercise programme including aerobic and resistance training in people with limited cutaneous systemic sclerosis

**DOI:** 10.1007/s10067-019-04921-7

**Published:** 2020-01-14

**Authors:** Alexandros Mitropoulos, Anil Gumber, Helen Crank, Mohammed Akil, Markos Klonizakis

**Affiliations:** 1 Centre for Sport and Exercise Science, Sheffield Hallam University, Sheffield, UK; 2 Centre for Health and Social Care Research, Sheffield Hallam University, Sheffield, UK; 3 Rheumatology Department, Royal Hallamshire Hospital, Sheffield, UK

**Keywords:** Digital ischemia, High-intensity interval training, Quality of life, Resistance training

## Abstract

**Objectives:**

It is suggested that exercise can improve the vascular function and quality of life (QoL) in people with systemic sclerosis (SSc), potentially offering clinical benefits to this population. Yet the feasibility of such an intervention remains untested. Therefore, the purpose of this study is to examine the feasibility of a combined exercise protocol (aerobic and resistance training) in people with limited cutaneous SSc (lcSSc).

**Methods:**

Thirty-two lcSSc patients (66.5 ± 12 years old) were randomly allocated in two groups (exercise and control group). The exercise group underwent a 12-week exercise programme, twice per week. All patients performed the baseline, three- and six-month follow-up measurements where functional ability, body composition and QoL were assessed. Participants’ experiences were explored through interviews.

**Results:**

Compliance was 92.6% with no dropouts. The individuals’ confidence to participate in the study’s exercise protocol for twice per week was 95%. The average value for the physical activity enjoyment scale was 103 ± 10 out of 119 (highest score). The mean values for the intention to engage in exercise twice per week were 6.4 ± 1 (likely) out of 7 (very likely). QoL for the exercise group showed to have a better life satisfaction, less anxiety and Raynaud’s phenomenon-accompanied pain.

**Conclusions:**

Our results suggest that a combined exercise protocol was feasible for people with lcSSc, with no adverse events, resulting in high adherence and low attrition rates, high enjoyment levels and intentions for future engagement to this exercise. Thus, the specific protocol is a safe adjunct therapy for people with lcSSc.

**Trial registration:**ClinicalTrials.gov (NCT number): NCT03058887, February 23, 2017, https://clinicaltrials.gov/ct2/show/NCT03058887?term=NCT03058887&rank=1

**Table:** 

**Key Points** *• High-intensity interval training in combination with resistance training constitutes a feasible exercise protocol for people with lcSSc*. *• Overall, the exercise programme demonstrated high adherence and enjoyment levels and low attrition rates.* *• The exercise protocol was proved to be safe with no adverse events for people with lcSSc*.

**Electronic supplementary material:**

The online version of this article (10.1007/s10067-019-04921-7) contains supplementary material, which is available to authorized users.

## Introduction

Systemic sclerosis (SSc) is an idiopathic systemic autoimmune disease characterized by an ongoing cutaneous and visceral fibrosis, vasculopathy and immunologic abnormalities [1]. It is a rare disease: There are almost 1200 new cases each year, with approx. 20,000 people living with the condition in the UK [2]. However, numbers will increase by 25% over the next 20 years due to the predicted growth and ageing of the population [2].

SSc can be either limited cutaneous (lcSSc) with skin involvement mainly limited to the hands and face, or diffuse cutaneous (dcSSc) with skin involvement proximal to the elbows and knees [3].

Blood vessels are directly affected by SSc and this has important ramifications on the quality of life (QoL) of patients. These vascular complications may progress to digital ulceration (DU) (approx. 55%); [4], gangrene and digital amputation [5]. DU in turn leads to hand function loss [6], and it is estimated that ≥ 50% of people with SSc have low or poor work ability [7]. SSc has the highest case-specific mortality and morbidity of any rheumatic disease with survival rates from diagnosis to 10 years being at 63% [8]. An important consideration when assessing the disease burden is that no cure exists. Pharmacotherapy (i.e. calcium channel blockers, phosphodiesterase inhibitors, prostacyclin analogs) is the main management option for this population: However, short-term (oedema, headaches, heart palpitations, dizziness and constipation) and long-term (heart dysfunction, increased cardiovascular risk) treatment side effects are frequent and should also be considered when deciding treatment plans. Therefore, alternative approaches with less side effects and cost implications are warranted.

A recent study from our research team [9] revealed that high-intensity interval training (HIIT) can improve microvascular function in the digital area of people with SSc. Considering the importance of a healthy skin microcirculation in avoiding digital ulcers, our findings suggest that there is an urgent need for further work in the field. Likewise, resistance training (RT) alone has shown significant improvements in the function of the vasculature [10], while a combination of aerobic and RT have shown both in the past [11] and recently [12] to significantly improve the vascular function and the microcirculation. However, the overall number of studies that have investigated the effects of RT on vasculature indicates remain. Moreover, to our knowledge the feasibility of implementing an exercise based on HIIT and RT as an adjunct therapy to standard pharmacotherapy is yet to be examined in people with SSc. In an era of limited financial and human resources, such a study will be important prior to the assessment of the clinical- and cost-effectiveness of the intervention in a large cohort.

Therefore, by applying a mixed-methods approach, this study investigated the feasibility of exercise to be performed by people with SSc using an established upper-limb HIIT protocol (arm cranking) and RT. This was assessed through adherence, compliance and attrition rates, exploration of enjoyment levels, assessment of exercise tolerance, number of adverse events and exploration of individual experiences. We also report on rates of screening, eligibility, and recruitment.

## Materials and methods

### Participants

We recruited thirty-two people (29 women, 3 men) with lcSSc, defined as per the American College of Rheumatology and European league against rheumatism criteria [13]. Eligible participants (Table 1) were recruited from the Rheumatology Department of the Royal Hallamshire Hospital in Sheffield and provided written consent to participate. The London - West London & GTAC NHS Research Ethics Committee (REC reference: 16/LO/0811) approved the protocol (IRAS project ID: 68096) and the study complies with the Declaration of Helsinki. Patients were randomly allocated (block randomisation) between the exercise (*n* = 16) and control (*n* = 16) groups. The randomisation was performed by an independent statistician. The allocated group was announced to both the principal investigator and the participant after the completion of the baseline measurements. All the pre- and post-intervention tests were performed at the same time of the day to minimize intra-day variability. An extensive methods section for our study has been published elsewhere [14]. A set of microvascular assessments was also undertaken but will be reported separately.

**Table 1:** Eligibility criteria

Inclusion criteria	Exclusion criteria
Patients diagnosed with limited cutaneous systemic sclerosis according to the 2013 ACR/EULAR criteria experiencing Raynaud’s phenomenon.	Patients with advanced pulmonary arterial hypertension or interstitial lung disease.
Men or women aged < 18 years old.	Patients who are diagnosed with another inflammatory condition.
Disease duration between 1 to 10 years.	Patients presenting myositis with proximal muscle weakness.
Patients should be able to perform exercise.	Patients with New York Heart Association class 3 or 4.
	Current smokers or people who stopped smoking within 4 weeks of health screening.
	Women who are currently pregnant.

*ACR*, American College of Rheumatology; *EULAR*, European League Against Rheumatism

The study has been registered in ClinicalTrials.gov (NCT number): NCT03058887. This work was conducted under a PhD program, supported by the National Centre of Sport and Exercise in Medicine and the Sheffield Teaching Hospital NHS FT [15].

### Exercise programme

The exercise group undertook twice-weekly supervised exercise sessions at three different sport venues: the (a) Centre of Sport and Exercise Science at Sheffield Hallam University, (b) Graves and (c) Concord sport centres in Sheffield.

### High-intensity interval training

Each session started with a 5-min warm-up on an arm crank (involving light aerobic exercise and gentle range of motion exercises). This was followed by HIIT for 30 s at 100% of PPO interspersed by 30-s passive recovery for a total of 30 min. At the end of the session, patients undertook a 5-min cool-down period, involving lower- and upper-limb light-intensity aerobic exercise and light stretching. Patients were wearing heart rate monitors throughout the exercise sessions. Heart rate and RPE, and affect (see below) were assessed at regular intervals throughout the supervised exercise session.

### Resistance training

With respect to the RT, patients performed five upper body exercises in a circuit row for three circles interspersed by 2–3 min. In between the exercises, 10 to 20 s were allowed for a safe movement from one exercise to the other. The intensity was kept to 10 maximum repetitions and weight adjustments were done to compensate for any strength improvements during the exercise intervention. The intensity was monitored using Borg’s scale [16] 6–20 point. The five RT exercises were as follows: (1) chest press with dumbbells on a 30° inclined bench, (2) arms lateral raise with dumbbells in a seated position, (3) biceps curl with dumbbells, (4) triceps extension on the pulley from a standing position and (5) handgrip dynamometer.

### Procedures

Reported baseline assessments undertaken at baseline included anthropometry, functional ability and quality of life. Thereafter, patients were randomly allocated to two groups (exercise and control group). The exercise group (HIIT and RT) performed a 12-week exercise programme whereas the control group did not perform the exercise programme. Both groups were followed up after a 12-week (3 months) and 24-week (6 months) period performing the same measurements as conducted at baseline.

To support the successful participation of our participants, we used our “six pillars of adherence” framework (based upon “social support”, “education”, “reachability”, “small groups intervention implementation”, “reminders” and “simplicity”), which we have used previously with excellent results in lifestyle interventions (i.e. over 90% of retention and 79% of completion); [17, 18].

### Study outcomes

#### Feasibility and acceptability outcomes

Recruitment rates were measured as rate of invited participants who were eligible and consenting. Acceptability of allocation was assessed by assessing the attrition rates and comparing the two groups and examining reasons for dropout. Suitability of measurement procedures was assessed by outcome completion rates and reasons for missing data. Attrition rate was defined as discontinuation of intervention and loss to follow-up measurement for all conditions. The acceptability of the exercise programme was evaluated by using session attendance and compliance data and participants’ feedback via one-to-one semi-structured interviews conducted with a subgroup of participants after the 3-month follow-up visit. Moreover, we assessed as measures for the acceptability of exercise, the participants’ enjoyment levels, intentions of engagement to exercise and task-self efficacy after the end of the exercise session at several time points during the exercise intervention. We also monitored the rate of perceived exertion and affect throughout each exercise session so as to document important information about the acceptability of exercise. The safety of exercise was assessed by exploring reasons for dropout from the exercise programme and the number and type of adverse events that occurred during the exercise intervention.

#### Quality of life

The EQ-5D-5L was the main outcome used to assess the patients’ QoL pre- and post-exercise intervention. The EQ-5D-5L is a generic measure of health state by considering five key dimensions of daily living (mobility, self-care, ability to undertake usual activities, pain, anxiety/depression) [19]. Participants were asked to describe their level of health on each dimension using one of five levels: no problems, slight problems, moderate problems, severe problems and extreme problems. Patients were also asked to rate their life satisfaction on a scale of zero to ten as well as to rate the RP pain during the last couple of weeks on a one to five ascending grading: not at all, slightly, moderately, severely and extremely. Digital ulcers and hospitalization for iloprost infusion and amputations were also recorded.

#### Functional ability test

The functional ability was assessed through a six-minute walking test (6MWT). Although the 6MWT lacks organ specificity in SSc, it can provide a valuable outcome parameter and thus, is suggested as a regular assessment in this clinical condition [5]. Patients were instructed to walk as far as possible back and forth on a 10-m corridor for 6 min. They were also instructed to slow down, stop and/or rest as necessary if they got out of breath or became exhausted, but to resume walking as soon as they felt able to. The laps and the total walking distance were recorded on a worksheet.

#### Exercise tolerance

The exercise tolerance of HIIT was assessed through measures that interpreted participants’ perception regarding the exercise intensity [16], the affect (Supplementary material Appendix A), the exercise task self-efficacy (Supplementary material Appendix B), the intentions (Supplementary material Appendix C) and the enjoyment (Supplementary material Appendix D). The above data was collected at the first and last exercise session each month in order to examine several time points during the exercise intervention. Specifically, the questionnaires were repeated at the 1st, 8th, 16th and 24th exercise sessions. The individual questionnaires and the time points that were incorporated during the exercise session are described in Jung et al. [20].

#### Interviews

Semi-structured interviews were conducted by AM in a randomised purposive sample of 12 patients from exercise (*n* = 6) and control (*n* = 6) group. The interviews were all held at the Sheffield Hallam University. HC and AM developed a semi-structured interview guide, which acted as a trigger and a motivation for further conversation. The interview guide was piloted in interview 1 and only minor changes were subsequently made. The guide is presented in Table 2. Each interview ended with the interviewer asking the patient if they wanted to make any additional comments not explored via the interview guide.

**Table 2:** Interview guide

Focus topic: Patients’ experiences of RP	Please, describe your feelings when RP attacks take place, symptoms, thoughts, when and how much do they occur.
Focus topic: Treatment of RP	Advice given by clinicians, how efficient is it, how satisfied are you, side effects from medical treatment.
Focus topic: Exercise intervention-study procedures	Please, describe your experiences regarding the study procedures, exercise intervention, effects upon RP and QoL, motivations from supervised exercise training, lifestyle changes. Your thoughts about exercise training and its potential benefits.

The interviews lasted between 15 and 20 min and were digitally recorded. The interviewer (AM) transcribed the recordings.

#### Anthropometry

The participant’s stature was measured using a Hite-Rite Precision Mechanical Stadiometer. Body weight (kg), body mass index (BMI), fat mass (kg) and lean body mass (kg) segmented in upper and lower limbs were assessed by using bio-electrical impedance analysis (In Body 720, Seoul, Korea). Participants’ demographic characteristics are illustrated in Table 3.

**Table 3:** Demographic data (means ± SD)

	Baseline Exercise (*n* = 16)	Baseline Control (*n* = 16)	Baseline Total (*n* = 32)
Age (years)	69.6 ± 11.4	63.6 ± 12.2	66.5 ± 12
Body weight (kg)	64.7 ± 10.2	72.2 ± 14.2	68.4 ± 12.7
Body mass index (kg/m^2^)	24.8 ± 3.1	26.6 ± 4.6	25.7 ± 4
Stature (cm)	161.5 ± 9	164.5 ± 6.1	163 ± 7.7
Disease duration (years)	8 ± 2	8 ± 2	8 ± 2
History of DUs (%)	30 ± 3	29 ± 5	30 ± 4
Clinical course of DUs*	1.2 ± 0.92	1.3 ± 0.81	1.2 ± 0.86
Raynaud’s treatment	9/16	13/16	22/32
Nifedipine	7/9	7/13	14/32
Sildenafil	2/9	6/13	8/32
Blood pressure treatment	8/16	7/16	15/32
Candesartan	3/8	3/7	6/32
Ramipril	5/8	4/7	9/32

*Mean number of DUs per patient per year during the last 5 years (2012–2017)

### Overall data analysis

We used rates of eligibility, recruitment, attrition, outcome completion, exercise adherence and adverse events to assess the feasibility and acceptability of the intervention. Enjoyment levels and intentions for exercise, as well as task self-efficacy of exercise are also presented. Individual’s experiences relative to the feasibility and acceptability of exercise are reported. Frequency counts and percentages were provided for categorical data. Continuous variables were summarized with descriptive statistics. All data analysis was conducted at the end of data collection, using SPSS software (version 23, IBM SPSS, New York, USA). Data are presented as mean ± SD. Interviews were analysed using framework analysis [21]. Analysis was aimed at describing the individual’s experience of exercise, searching for common, recurrent patterns and also identifying an understanding of participant experiences that might explain the feasibility and acceptability of exercise. The coding framework that was used for the interview analysis is of a deductive approach, framing the analysis within a priori topic guide, yet data were borne out of original transcripts from the interviews [22].

The sample size calculation for our study estimated the critical metrics needed to assess the feasibility of conducting the definitive study, with sufficient precision [14]. The critical metrics are the consent rate (i.e. the proportion of eligible patients who consented to participate and be randomised), improvement in QoL as well as compliance with treatment, and attrition rates. Fifteen patients in each group (*n* = 30 in total) provided a sufficiently precise (within 15 percentage points for a 90% confidence interval) estimate of the proportion willing to be randomised, assuming 35% intention to be randomised.

## Results

### Recruitment rates

**Fig. 1:**
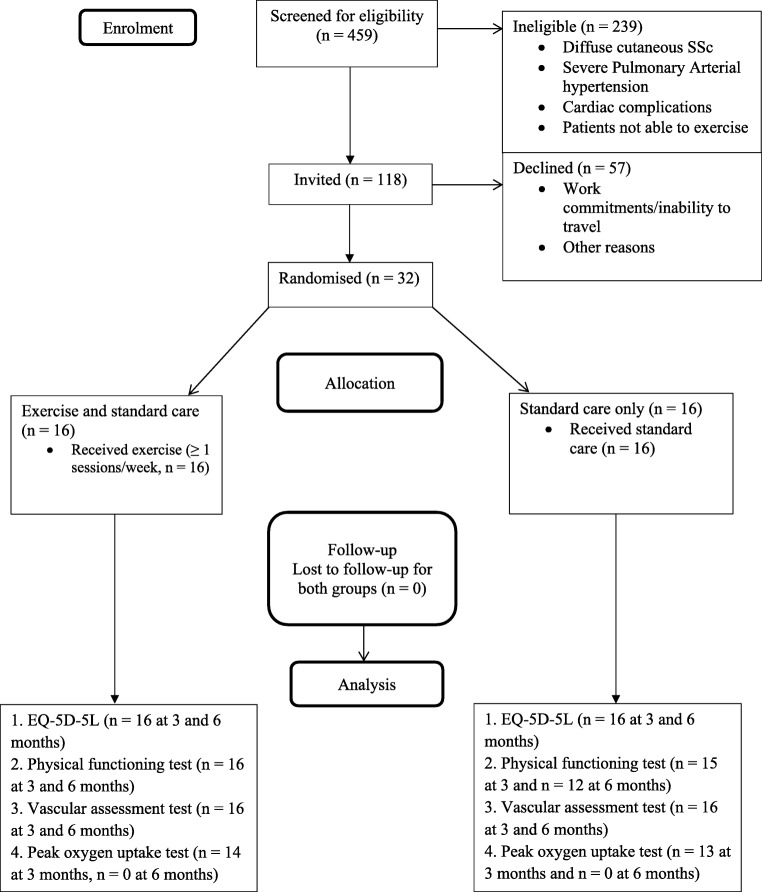
Flow of participants through the trial

Figure 1 shows the flow of participants through the trial. Recruitment took place between January 2016 and December 2017. Of 459 people with SSc screened for participation, 220 met eligibility criteria and 118 were invited. From those invited, 32 were recruited (3 men and 29 women), giving eligibility and recruitment rates of 47.9% and 27.1% respectively.

### Feasibility of exercise

Compliance to the twice weekly, 12-week exercise programme was 92.6% with no dropouts. The average percentage of peak HR (% HR_peak_) for the aerobic part of the training was 89.6% ± 4.5. The average RPE and affect during both the aerobic and resistance exercise was 13 ± 1 (“somewhat hard”) and 3 ± 1 (“good”), respectively. The average value for the physical activity enjoyment scale was 103 ± 10 out of 119 (highest score). The mean values for the intention to engage in exercise twice and thrice per week were 6.4 ± 1 (likely) and 5.3 ± 2 (slightly likely) out of 7 (very likely). The individuals’ confidence to participate in the study’s exercise protocol for once and twice per week was 95% and 80% for three times per week out of 100%. No exercise-related complications were reported.

### Interview findings - quality of life

The interview findings are presented in Table 4. QoL improved significantly in several domains for the exercise group: More specifically, it appeared to have better life satisfaction (9.25 ± 0.9), less difficulty to perform the usual activities (1.63 ± 0.7), less anxiety (1.06 ± 0.3), and less Raynaud’s phenomenon-accompanied pain (2.19 ± 1) than the control group, following the exercise intervention. Benefits were maintained 6 months after baseline as well (Table 5).

**Table 4:** Interview main findings

Theme	Main finding	Quotes of participants
Experiences of Raynaud’s phenomenon (Theme 1)	During an RP attack, individuals find it hard to carry on with their daily activities.	“When I do have an attack of the Raynaud’s my fingers do go numb…It is very painful as well when they are warming back up so I cannot do anything until this comes back to normal.” (SSc009-CG).
Experiences of Raynaud’s phenomenon (Theme 1)	Activities of daily living are restricted and as such quality of life is adversely affected particularly in the winter.	“Usually my finger ends can become quite painful and particularly in cold weather and even if I go out to put something in the bin I would put a jacket and gloves on... So everything has to be carefully though about before I do any jobs.” (SSc013-CG).
Participants positive experiences of study intervention (Theme 2)	Participants in the exercise group appeared to have enjoyed the HIIT exercise training and this fostered intention to engage in exercise after the end of the study.	“I quite enjoyed the regime and doing it”, (SSc005-EG). “Had I the facilities and the opportunity I would continue to do that”, (SSc002-EG).
Participants positive experiences of study intervention (Theme 2)	Supervision in the exercise sessions gave participants reassurance about the safety of engaging in exercise and it also proved to be one of the key factors that participants enjoyed and valued about the exercise intervention.	“I know people who go to gyms and have a personal trainer and I was though “how pretentious” they are… But actually, really does encourage you…it also makes you more confident… and you are not worried that you are gonna…strain something.” (SSc002-EG).
Participants positive experiences of study intervention (Theme 2)	Supervision helped the participants to both adhere to the exercise programme, as well as the exercise protocol*.*	“Oh that was good. It definitely did because as I said it was like having your own personal trainer.” (SSc010-EG). “On your own devices you do not do things the same do you? But if you know that there is someone there to say “did you do it?” and you cannot say no then you do it, do not you? Because you were there all the time I could not stop doing it could I?” (SSc018-EG).
Participants positive experiences of study intervention (Theme 2)	Participants reflected that exercise improved their QoL through specific mechanisms relating to physical, mental and social well-being.	“Exercise improved my fitness and socialising.” (SSc005-EG). “After exercise, I feel more happy and more energetic, I feel stronger” (SSc010-EG).
Barriers to exercise (Theme 3)	One of the main barriers mentioned by participants was access to the exercise venue; the transport and the travelling time that it involved.	“The main thing for me is transport. Where I live a couple of years ago we had a descent bus service now Sheffield it’s just changed all its buses and it is just helpless…” (SSc002-EG).
Barriers to exercise (Theme 3)	Attendance depended not only on the distance and travelling time but also on the time slot that the exercise sessions could be performed. When travelling time was reduced, participants found it was more feasible to attend the sessions.	“Certain hours trying to get in Sheffield on a rush hour takes twice as long as normal... On my days off it really does not make that much difference…” (SSc016-CG). “When I went to Graves sport centre (outskirts of Sheffield) I found it easier.” (SSc011)
Barriers to exercise (Theme 3)	Another important barrier for this clinical population was the impact of bad weather. Participants did not feel confident to go out in cold weather.	“No in winter. I think I would struggle due to the cold.” (SSc011-CG). “if I am standing any length of a time at the bus stop I can get very cold indeed well I would not like rely on coming on buses in winter time.” (SSc013).

**Table 5:** Quality of life outcomes

	Exercise (*n* = 16)			Control (*n* = 16)		
	Baseline	12 weeks	24 weeks	Baseline	12 weeks	24 weeks
Life satisfaction	8.13 ± 2.2	9.25 ± 0.9*	9.38 ± 0.9**	7.31 ± 1.4	7.33 ± 1.8	6.83 ± 2
Mobility	2.0 ± 1	1.63 ± 1	1.75 ± 0.7	1.81 ± 0.8	2.07 ± 1	2.17 ± 0.9
Self-care	1 ± 0	1.19 ± 0.5	1.06 ± 0.3	1.25 ± 0.8	1.6 ± 1.1	1.42 ± 1.2
Usual activities	1.69 ± 0.8	1.5 ± 0.7*	1.63 ± 0.7*	1.88 ± 1	2.33 ± 1	2.42 ± 0.9
Pain	2.44 ± 1	1.81 ± 1	1.75 ± 0.7*	2.19 ± 0.8	2.47 ± 1	2.42 ± 0.9
Anxiety	1.38 ± 0.6	1.06 ± 0.3*	1.13 ± 0.3*	1.69 ± 0.7	1.8 ± 1.2	1.83 ± 0.9
Raynaud’s pain	2.19 ± 1.2	2.19 ± 1*	2 ± 0.9*	2.5 ± 1.1	3.07 ± 1	2.83 ± 0.9

*p < 0.05 and **p < 0.001 compared to control group

### Digital ulcers

The exercise group did not present any digital ulcers (DUs) throughout the 6-month period of the study whereas the control group presented five incidents (32% of the control group) of DUs and four hospitalisations for iloprost infusion.

## Discussion

This is a feasibility study, an important step prior to the assessment of the clinical- and cost-effectiveness of the intervention in a large cohort. The results of our primary outcomes (e.g. feasibility and acceptability outcomes, quality of life, exercise tolerability and interviews) support the progression to a definitive, multi-centre trial with a larger cohort of people with SSc.

### Feasibility outcomes and individuals’ experiences of exercise intervention

Evidently, the high rates of compliance and retainment to the implemented exercise programme (92.6%) is an encouraging sign of the acceptability of our novel intervention, aiming at people with SSc. Participants appeared to enjoy the exercise sessions and were motivated to adhere to the exercise programme. Supervised exercise was considered by most of our participants as a safe and educating approach, allowing our participants to gain a greater degree of confidence in exercising, opening the way for self-managing their sessions in the future. Therefore, it could be suggested that supervised exercise can be a key element for a definitive exercise programme. This is in agreement with findings from other similar, supervised exercise programmes in groups with vascular clinical manifestations (e.g. venous leg ulcers; 18). In contrast, unsupervised home-based exercise programmes in people with idiopathic pulmonary fibrosis demonstrated significantly low levels of exercise attendance and limited improvement [23–25].

The exercise programme stressed the cardiovascular system moderately (aver. HR_peak_ = 89.6%) and thus, the RPE was also relatively low (13 “somewhat hard”, Borg scale) and the mean affect was reported as good throughout the whole exercise session (+ 3 “good”). The average enjoyment score of the exercise sessions was also high (103 ± 10). From a physiological perspective, the enjoyment of the exercise could be explained by the low levels of lactic acid production that a short HIIT protocol is able to induce [9, 25, 26]. Moreover, the participants did state that they enjoyed the exercise sessions attributing this feeling to the supervised training, to the welcoming environment and to the tangible improvements in their breathing and fitness status (Theme 2). Another important finding is the high score in the task-self efficacy questionnaire of 95% and 80% for two and three bouts per week, respectively. This shows the feasibility of our exercise protocol and the possibility to increase the training dose (three times per week) giving that it might induce greater improvements.

Participants’ intentions towards engaging in our exercise protocol twice and thrice per week were positive throughout the exercise programme. Participants responded that it would be likely for them to engage in our exercise protocol at least twice per week (6.4 ± 1) and slight likely to engage at least three times per week (5.3 ± 2). Moreover, participants’ perspective for exercise is that it contributes to the overall wellbeing by improving the fitness and social status, mental health and forms a positive approach towards life in general (Theme 2).

It is important to note that none of the participants mentioned exercise sessions’ duration as a barrier, which further highlights the feasibility of our exercise protocol to be implemented in people with SSc. The main two barriers were venue accessibility and weather (Theme 3). Participants can find it very challenging to travel to central exercise locations from the city outskirts: This is a significant barrier which requires strong motivation to sustain study participation. In our study, we offered a community-based programme across several sites, minimising travelling time for participants. This would be important feature for future interventions. Weather constituted another key barrier for our participants: Nevertheless, a short travelling time would increase the possibility of participation (Theme 3). In our study, we attempted to overcome participation barriers, by offering also a high number of available time slots to accommodate participants’ preferences and fit with their daily schedule. Thus, the high rates in attendance and engagement can be attributed to the short travelling time to exercises venues and the wide range of the exercise sessions’ availability. The same approach should be followed in a future, definitive trial as well.

#### Quality of life

People with SSc’ QoL is adversely affected by RP attacks which induce numbness, pain and restrict individuals from performing their daily activities. An RP attack may last up to several hours and the most people with SSc in our study reported that they are unable to perform their activities unless they perceive blood flow that comes back to normal (Theme 1). The RP symptoms such as very cold hands or hand disfigurement can affect the social life in people with SSc acting as a psychosocial burden/anxiety (Theme 1).

The current study’s QoL findings indicate that life satisfaction and RP-related pain were improved significantly in the exercise HIIT group compared with the control group, which aligns with the findings of our previous pilot trial [9]. Moreover, people with SSc that took part in our exercise intervention had less anxiety and were more readily able to perform their usual activities compared with the control group. Noticeably, these positive findings for the exercise group were maintained 3 months after the completion of the exercise intervention compared with the control group. Therefore, our exercise protocol seems capable of improving QoL in this patient group.

#### Clinical outcome

Our study demonstrated that a combined exercise programme is feasible to be implemented in people with SSc. In addition to that, we observed a beneficial effect of exercise on DUs which negatively affect QoL in people with SSc. Specifically, 32% of the control group developed DUs and most of them required hospitalisation to heal, whereas the exercise group had no incidents of DUs, even 3 months after the cessation of the exercise programme.

DUs are common in SSc and approximately half of patients reporting a history of DUs [4, 27–29] and ~ 10% presenting current DU [4, 30]. Often, DUs are presented early in the disease [27]. Patients with a shorter duration between the first and the second DUs (especially if the second is within 2 years) have an increased (yearly) DU burden [27]. About one- and two-thirds of people with SSc develop recurrent DUs [27, 28, 31]. DUs often involve both hands with multiple fingers [6, 27] and DUs per episode [27, 31]. The healing of DUs is often slow, specifically if there is underlying calcinosis, and can be related to underlying bone infection [32].

The indicated clinical benefits of the proposed exercise intervention, in relation to DU occurrence, strengthen the suggestion that there is a need for a multi-centre clinical trial which would assess its effectiveness on DUs and other clinical components.

## Conclusions

Our findings suggest that an exercise programme combining HIIT and RT was feasible for people with SSc, resulting in high adherence and low attrition rates, high enjoyment levels and intentions for future exercise engagement. Our participants felt comfortable and capable of performing our protocol without experiencing any adverse events.

Based on our study findings, we would recommend that such a programme would be better delivered in the community: this is definitely the patients’ preference as it would eliminate one of the main barriers to exercise participation, which is the travelling time and transportation.

We also believe that the addition of another exercise session per week (three times/week) would induce greater results. However, a community-based programme needs to be pragmatic and thus, two times per week is the recommended feasible training frequency as our study indicated.

Finally, it was also very encouraging to see that our intervention elicited improvements in QoL, supporting also the prevention of clinical manifestations such as digital ulcers. This will need to be proven in a definitive trial, which is the next logical step, in this line of research.

### Limitations

We only included people with limited cutaneous systemic sclerosis. In this population, changes in skin thickness are little over time, when compared with diffuse cutaneous systemic sclerosis [33]. Thus, and as it is a common practice in the UK national healthcare system clinics to assess the modified Rodnan skin score only in patients with dcSSc, this measurement was not collected and included for the purposes of this study. However, considering the clinical facts [33], we do not feel that this choice has affected our findings or conclusions.

## Electronic supplementary material

ESM 1 (DOCX 35.4 kb)

ESM 2 (DOCX 40 kb)

ESM 3 (DOCX 34.7 kb)

ESM 4 (DOCX 44.5 kb)

## Data Availability

Relevant files of this work will be shared on request.

## Abbreviations

6MWT: six-minute walking test ACR American College of Rheumatology BMI body mass index CG control group dcSSc diffuse cutaneous systemic sclerosis DUs digital ulcers EG exercise group EULAR European League Against Rheumatism HIIT high-intensity interval training HR heart rate lcSSc limited cutaneous systemic sclerosis LDF laser Doppler fluximetry QoL quality of life RP Raynaud’s phenomenon RPE rate of perceived exertion RT resistance training SSc systemic sclerosis
